# Family intimacy and adolescent peer relationships: investigating the mediating role of psychological capital and the moderating role of self-identity

**DOI:** 10.3389/fpsyg.2023.1165830

**Published:** 2023-06-29

**Authors:** Xin Zhou, Jin Huang, Sushu Qin, Kangsheng Tao, Yumei Ning

**Affiliations:** ^1^School of Humanities and Education, Enshi Vocational and Technical College, Enshi, China; ^2^School of Economics and Management, Enshi Vocational and Technical College, Enshi, China; ^3^School of Economics and Management, Hubei Minzu University, Enshi, China; ^4^School of Business Administration, Zhongnan University of Economics and Law, Wuhan, China; ^5^Business School, Yulin Normal University, Yulin, China

**Keywords:** adolescent peer relationships, family intimacy, psychological capital, self-identity, ecological systems theory

## Abstract

According to existing research, family intimacy affects the formation of peer relationships among adolescents; Parent–child relationships may influence children’s relationships with peers, but the mechanism of its influence is still unclear due to the uncertainty of its effect. According to the ecological systems theory, this study examines how family intimacy affects adolescent peer relationships through psychological capital and how self-identity moderates this effect. These hypotheses were tested based on a survey of 414 adolescents, which showed that family intimacy positively affects adolescent peer relationships; The relationship between family intimacy and adolescent peer relationships is mediated by psychological capital; Self-identity positively moderates the direct effects of family intimacy and adolescent peer relationships; Self-identity not only positively moderates the direct effect of psychological capital and adolescent peer relationship, but also positively moderates the indirect effect of family intimacy on adolescent peer relationship through psychological capital. This study provides new perspectives on the relevant mechanism of family intimacy and adolescent peer relationships.

## 1. Introduction

For decades, peer relationships have been considered by scholars to be one of the most important social relationships for adolescents. Peer relationship is a kind of interpersonal relationship developed by individuals of similar age or psychological development levels in the process of communication and cooperation. It is regarded as an important indicator to effectively measure the ability of adolescents to adapt to the social environment and cope with difficulties (Rubin et al., 2013). As non-kinship relationships, the development of adolescent peer relationships is affected by many different factors in family, school, and society (Ladd et al., 2008; Zhu et al., 2022). Adolescents who are unable to effectively establish positive peer relationships may experience a decrease in their ability to accurately assess the value of relationships (Rosenbach and Renneberg, 2014; Long et al., 2021), and even show withdrawal and avoidance of future interpersonal communication and social activities (Molden et al., 2009; Haddow et al., 2021). Having good peer relationships plays an important role for individuals in adolescence. On the one hand, it can help adolescents develop positive interpersonal relationships and adapt to complex social situations, which directly impacts adolescents’ self-identity; on the other hand, it can be a valuable source of emotional support for adolescents (Crosnoe and Johnson, 2011). Ecological systems theory suggests that everyone lives in a specific environment. Family and peer relationships are the most important microsystems for adolescents (King et al., 2016; McMahon et al., 2020). It has been found that family intimacy affects adolescent peer relationships (Zemp et al., 2018; Noonan and Pilkington, 2020). The influential mechanism of the complex relationship between family background and peer relationships needs further investigation. Therefore, it is meaningful to study the influence mechanism of family intimacy on peer relationships, which can improve the level of positive peer interaction among adolescents.

How to establish positive peer relationships has become a focus of attention in education, psychology, medicine, and other fields. Based on Bronfenbrenner’s (1979) ecological systems theory, Peer relationships have been shown to be significantly affected by parental conflict (Mcdowell and Parke, 2005; Racz et al., 2017), family socioeconomic status (Hjalmarsson, 2018; Bukowski et al., 2020), and parent–child communication (Runcan et al., 2012; O’Mara and Schrodt, 2017). Positive peer relationships among adolescents can also be affected by school factors in addition to family factors, such as academic performance (Horoz et al., 2022), school exclusion (Gabbiadini and Riva, 2018; Andrews et al., 2019), and school belonging (Demanet and Van Houtte, 2012; Fong Lam et al., 2015). Despite this, most of the data to date show that research has focused primarily on peer relationships as a factor in adolescents’ psychological development and social adjustment, while the exploration of family intimacy in adolescents’ positive peer relationships has been very limited. The factors of family intimacy on the establishment of adolescent peer relationships and the complex mechanism of action between these factors should be further explored in future research to provide theoretical guidance for improving the establishment of positive adolescent peer relationships.

This study examines the current body of research on adolescent peer relationships, focusing on three key areas. First, this study aims to investigate the influence of family intimacy on adolescent peer relationships. Family intimacy can reflect the degree of emotional connection that an individual feels with other family members and is an important indicator reflecting the emotional relationships and positive family atmosphere among family members (Lambert et al., 2010; Umberson and Thomeer, 2020). When adolescents have someone who supports them emotionally, they feel peaceful in the face of stress (Romano et al., 2021), and their extraversion and emotional stability are also more obvious. A review of previous literature indicates that most existing research on family intimacy and adolescent peer relationships follows three paths: behavioral problems (Cummings et al., 2015; Weymouth et al., 2016), psychological development (Sabatelli and Anderson, 1991; Gregory and Sadeh, 2012), and social skills (Okuno et al., 2016; Curran et al., 2021). Peer groups are not only just a source of motivation for adolescent development, but also an important driving force for personal social–emotional development, positive mental health development, and even academic development (McFarland et al., 2014). As a result, it is imperative to examine the impact of family intimacy on adolescents’ peer relationships.

Second, the study explores how psychological capital mediates the relationship between family intimacy and adolescent peer relationships. In ecological systems theory, individuals and the factors around them are interdependent and mutually constrained. Psychological capital refers to positive psychological resources. It is the positive psychological state shown by individuals when they get along with their families and peers in their growth environment (Çavuş and Gökçen, 2015), revealing the interaction process between individuals and other social factors. It includes four characteristics: psychological resilience, optimism, sense of efficacy, and hope. When these characteristics are combined, they have a more positive influence on individuals (Grover et al., 2018). Having close family relationships can contribute to the development of positive psychological states in an individual (Luthans et al., 2005; Waters et al., 2022). Psychological capital has been shown to positively influence individual attitudes and behaviors (Hobfoll, 2002; Kun and Gadanecz, 2022). Psychological capital, as the mechanism of family intimacy in adolescent peer relationships, deserves further investigation.

Finally, self-identity is examined in this study as a moderating factor in the relationship between family intimacy and adolescent peer relationships as well as between psychological capital and adolescent peer relationships. According to the ecological systems theory, adolescents have strong family emotional support, which will have an important impact on their peer communication and social adaptation (Luthans et al., 2007). However, not all adolescents are like this. The growth of adolescents is a process of self-cognition and self-development, which will continue to change with different ages and experiences (Korol, 2021). The moderating effect of self-identity on adolescent psychological adaptation has been confirmed. When faced with stress and difficulties, A low sense of self-identity among adolescents increases the likelihood of depression and withdrawal (Toti et al., 2020). Different levels of self-identification will affect adolescents’ different psychological states, thus affecting adolescents to make a more comprehensive and objective understanding of themselves (Dugarova et al., 2020; Rizki and Keliat, 2021). However, previous studies have rarely addressed this effect. Therefore, this study proposes that self-identity is a moderating variable between family intimacy and psychological capital.

This study makes three contributions. Firstly, it examines the influence of family intimacy on adolescent peer relationships through the lens of ecological systems theory; Secondly, this study explores the mechanism of adolescents’ psychological capital affecting family intimacy and peer relationships from the perspective of ecological systems theory; Thirdly, our research makes a contribution by exploring the role of self-identity as a moderate between family intimacy and adolescent peer relationships, as well as between psychological capital and adolescent peer relationships. This study emphasizes the importance of valuing one’s sense of self-identity and actively cultivating it, as it can promote the development of healthy peer relationships during adolescence.

## 2. Theory and hypothesis

In this study, the role of family intimacy in peer relationships among adolescents is supported theoretically. According to the ecological systems theory, the relevant conceptual research framework and hypothesis are proposed in this study. Influencing factors will be explored through empirical research using the conceptual framework.

### 2.1. Ecological systems theory

The ecological systems theory was developed by Bronfenbrenner (1979), which revealed the interplay between different systems, and the pathways by which family intimacy affects adolescent peer relationships can be better understood when this interaction is explicitly considered. Some studies believe that the social environment will affect the physical and psychological characteristics of individuals, and all environments from family to economy and politics have become part of the life development process (Ceci, 2006; Ozaki et al., 2020). The bioecological model proposed by Bronfenbrenner is one of the most cited theories in related fields (Crawford, 2020; Yu et al., 2021). It has expanded from research on personal development to research on the balance of family, school, and social development (Dobson and Douglas, 2020; El Zaatari and Maalouf, 2022).

In addition to ecological systems theory, some studies have used attachment theory (Mathes et al., 2020), cognitive-situation theory (Siffert and Schwarz, 2011), and perfectionism theory (Stumpf and Parker, 2000) to examine how family intimacy affects adolescent peer relationships. Adolescents’ behavior and psychological development are strongly influenced by their family environment, which is the basic unit of society. Some studies have found that the development of adolescents’ poor peer relationships, low academic performance, and poor social skills are inextricably linked to family factors such as weak intimacy among family members, inappropriate emotional expression, and improper parenting styles. Many problem behaviors of adolescents do not originate from themselves but are affected by the family environment (Buehler, 2020; Scully et al., 2020). According to Ruzek et al. (2016), Social adjustment and mental health of adolescents are heavily influenced by peer relationships. It requires adolescents to use self-identity to actively coordinate the relationship between individuals, families, and peers. Living in an environment with close family intimacy, adolescents are more likely to form self-identity. At the same time, psychological capital may enable individuals to perceive and evaluate the availability of their positive mental states. In the context of psychological capital, a better understanding of the mechanism by which external family environmental factors affect the internal psychological state and behavioral performance of adolescents. Guided by ecosystem theory, this paper establishes a theoretical framework to explain the mechanism by which family intimacy affects peer relationships, and demonstrates the effect of psychological capital and self-identity under the above mechanism.

### 2.2. Research hypothesis

**Figure 1:**
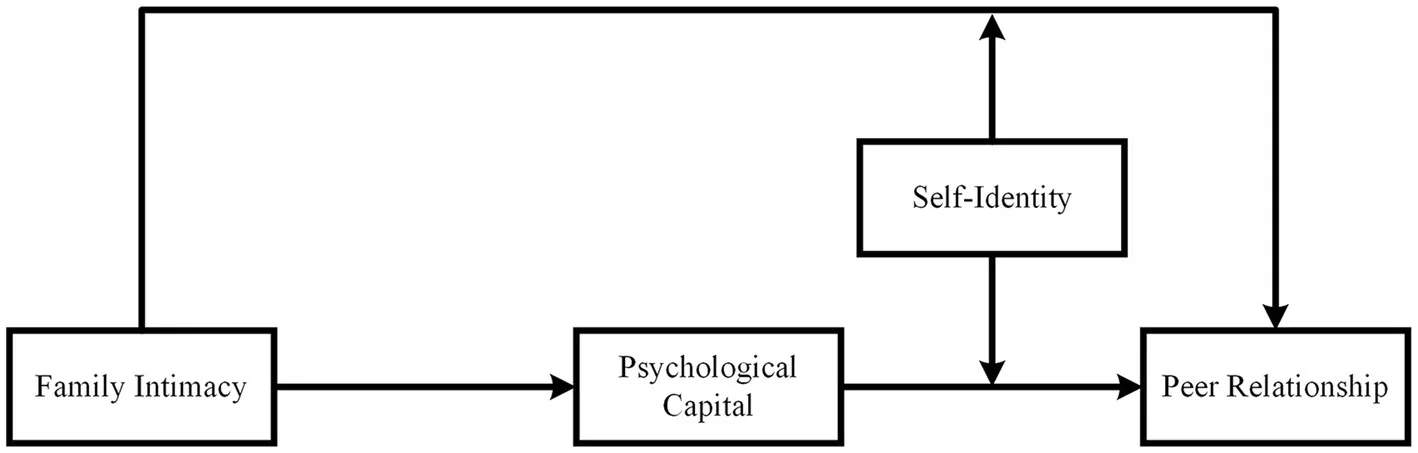
Theoretical model.

The impact of family intimacy on adolescent peer relationships is primarily examined through the following hypothesis. Psychological capital and self-identity are introduced as mediating variables and moderating variables in the analysis of the effects of family intimacy on adolescent peer relationships in adolescents. Figure 1 below shows the research framework.

#### 2.2.1. Family intimacy and adolescent peer relationships

Sociology believes that interpersonal relationship is the direct psychological connection established by people in communication. The intimacy and adaptability of the family are indicators to measure the family function and the overall operation of the family (Zhang and Wang, 2020). According to social learning theory, adolescents will apply the imitated parental mode of communication to their peer communication (Chuang, 2021). Existing research indicates that close parent–child relationships are beneficial for adolescents’ interpersonal skills and social adjustment (Lindell et al., 2021).

On the one hand, empirical studies have shown that in the family context, establishing an emotional connection between parents and children refers to an intimate relationship between them, and has an optimization and promotion effect on adolescents’ social cognition and interpersonal relationships. Adolescents living in low family intimacy environments have higher odds of feeling lonely and alienated in social interactions, which leads to anxiety and negative emotional states and interferes with adolescents’ interpersonal communication and daily learning and living (Martyn et al., 2009). On the other hand, research confirms that good family intimacy and positive psychological states of adolescents can influence and interact with each other (Cassels and Wilkinson, 2016). Relevant researchers have also studied the mechanism of family intimacy and adolescent peer relationships from the aspects of family-rearing style, evolutionary psychology, and social cognitive development. This leads to the following hypothesis:

*H1*: Family intimacy positively affects adolescent peer relationships.

#### 2.2.2. Family intimacy and psychological capital

The close family environment can promote the formation of individual positive psychological capital (Peterson et al., 2008; Xu et al., 2022). First, different parenting styles and communication styles of parents will lead to differences in children’s ability to get along with peers and adapt to socialization from the family environment with which they are most closely connected (Gimenez-Serrano et al., 2022). Second, family intimacy influences adolescents’ social, psychological, and behavioral development to some extent (Haggerty et al., 2022). Adolescents who live in a healthy family environment also score higher on the four elements of psychological capital. They can be more optimistic and positive in the face of stress and negative emotions (optimistic), have more hope and confidence in the future (hope), have a higher self-efficacy in coping with negative motivation (self-efficacy), and have stronger psychological resilience in the face of external stimuli (psychological resilience) (Zeng et al., 2021). This leads to the following hypothesis:

*H2*: Family intimacy positively affects psychological capital.

#### 2.2.3. Psychological capital and adolescent peer relationships

Psychological capital can provide individuals with important protective resources and cognitive resources for social adaptation and creative activities (Luthans et al., 2007; Poots and Cassidy, 2020). The internal and external resources possessed by individuals with high psychological capital will encourage individuals to engage in more positive behaviors and make more positive attributions when faced with changes in the external environment, pressure, and failure (Luthans et al., 2005). Meanwhile, individuals with higher psychological capital also have a better positive state (Avey et al., 2008; Lei et al., 2020), and it is easier for them to obtain positive energy supplements from the external world when dealing with risks (Datu et al., 2016). Psychological capital has been shown to influence behaviors, attitudes, studies, and peer relationships in positive ways (Newman et al., 2014). In summary, adolescents with higher psychological capital also tend to have more positive peer relationships with their peers. This leads to the following hypothesis:

*H3*: Psychological capital positively affects adolescent peer relationships.

#### 2.2.4. Family intimacy, psychological capital, and adolescent peer relationships

Adolescents tend to establish equal peer relationships with non-authoritative individuals, which is different from vertical relationships such as parent–child relationships and teacher-student relationships. Interpersonal relationships will change to some extent as individuals grow. When entering early adulthood, adolescents will hope to gain more autonomy and independence from their parents, and the importance of peer relationships will also become more important (Reavis et al., 2015). Adolescents living in an environment with closer family intimacy will develop a more wholesome personality and better peer relationships. For adolescents who are at the stage of vertical relationships to parallel transformation relationships, the influence of peer relationships on their healthy psychological condition, good mental condition, and positive social adaptability is becoming more and more important (Oberle et al., 2018). The socialization development, personality formation, and peer relationship establishment of adolescents are inseparable from the influence of family intimacy (Dunn, 2004). Studies have found that adolescents who have been in an environment with long-term unbalanced parental relationships and family intimacy have lower levels of interpersonal security, weaker interpersonal skills, and are more prone to negative emotions. Close family intimacy is significantly positively correlated with interpersonal skills (Kuo et al., 2007). However, family is one of the microsystems that affects individuals, and family intimacy does not directly affect the establishment of positive adolescent peer relationships. A high level of psychological capital contributes to well-being, emotional commitment, organizational identity, and interpersonal communication (Bea and Chang, 2015). Reasonable use of positive psychological capital is more conducive to adolescents mobilizing other resources to regulate the pressure in the process of growth, cope with negative emotions and improve peer communication skills (Newman et al., 2014). This leads to the following hypothesis:

*H4*: The effect of family intimacy on adolescent peer relationships is mediated by psychological capital.

#### 2.2.5. The moderating effect of self-identity

Self-identity was introduced to the field of psychology by Erikson, an American psychologist (Ochse and Plug, 1986). Erikson divides life into eight stages, each stage has different contradictions, and individuals grow accordingly in the process of resolving the contradictions at different stages. The primary task of individuals in adolescence is the establishment of self-identity, which is dynamic and continuous self-knowledge, self-evaluation, and self-identity of individuals in the social environment structure (Rodríguez-Meirinhos et al., 2020). The micro-system that individuals first contact is family. From family relationships, individuals begin to understand themselves and form a preliminary sense of self-identity. Having a strong sense of self-identity can correctly recognize the difficulties and challenges in their social environment and help them make adaptive responses. Higher self-identity is linked to better mental health (Sánchez-Miguel et al., 2017). Adolescents with high self-identity are more optimistic and outgoing than those with low self-identity, and their psychological status is also healthier and more mature. To some extent, self-identity has a predictive effect on adolescent mental health (Dugarova et al., 2020). In addition, when adolescents have a higher sense of self-identity, their self-control and self-regulation abilities are also stronger, which is conducive to the establishment of adolescent peer relationships and the optimization of adolescent social development (Huang et al., 2021). Research confirms that positive correlation between adolescent self-identity and friendship quality, and at the same time, the model suggests that self-identity moderates the relationship between family intimacy and peer relationships among adolescents. This leads to the following hypothesis:

*H5*: Self-identity moderates the relationship between family intimacy and adolescent peer relationships. When adolescents have a high sense of self-identity, the positive relationships between family intimacy and adolescent peer relationships is stronger.

Studies on psychological capital have shown that adolescents experiencing family risks do not mean that they will have mental health problems, and certain personal qualities or environmental factors will help adolescents overcome adverse influences (Wright et al., 2013). Luthans et al. (2005) linked growth in self-identity to psychological abilities. As an internal force, positive psychological capital can help individuals enhance their ability to overcome difficulties and pressures, and at the same time improve their ability to adapt to society (Chen et al., 2022). This leads to the following hypothesis:

*H6*: Self-identity moderates the relationship between psychological capital and adolescent peer relationships. The higher the self-identity of adolescents, the stronger the impact of psychological capital on adolescent peer relationships.

With a high level of self-identity is beneficial for enhancing the relationship between psychological capital and adolescent peer relationships. Furthermore, Erikson (1994) believes that psychological capital is affected by personal self-identity to some extent. We further believe that Self-identity can be used to alleviate the impact of family intimacy on adolescent peer relationships through psychological capital. In addition, since the mechanisms through which family intimacy acts on adolescents’ peer relationships may vary according to the level of self-identity, we believe that under a high level of self-identity, family intimacy has a greater impact on psychological capital, which will greatly improve the establishment of peer relationships among adolescents. Therefore, we combined Hypothesis 4 and Hypothesis 6, and then came up with Hypothesis 7:

*H7*: Self-identity positively moderates the mediating effect of psychological capital, and the mediating effect is enhanced when self-identity is high.

## 3. Methods and procedures

This research mainly explores the mechanism of family intimacy in adolescent peer relationships and has no unethical conduct in the course of the research. The data collected in this research process is in an anonymous form, and the filling of the questionnaire follows the voluntary principle; therefore, according to the requirements of local laws, regulations, and institutions, this research does not require ethical approval and consent.

### 3.1. Data collection

The data collected through the questionnaire comes from different schools, different grades and different individuals. Before filling out the questionnaire, the purpose and use of collecting the questionnaires have been explained in detail to students and their head teachers, and the consent and support of the head teachers have been obtained. This study does not involve human clinical trials, and the questionnaires are filled out voluntarily and collected anonymously. The students themselves completed questionnaires on family intimacy, psychological capital, self-identity, and quality of friendships. In order to ensure that the data and questionnaire are valid and rational, this study focused on individual-level measurements and analyses, this study selected two schools in China (Enshi Vocational and Technical College and Hubei Minzu University) in March 2021 for a pre-survey. We invited some students from two schools to conduct questionnaire tests and interviews. The results of the questionnaire survey conducted among people with different levels of education did not show a significant difference. According to the respondents’ suggestions, the questionnaire was appropriately revised based on their feedback. Before the official distribution of the questionnaire, we got in touch with Hubei Minzu University, Zhongnan University of Economics and Law, Enshi Vocational and Technical College, Lichuan NO. 1 Senior High School, Xuanen NO. 1 Senior High School, Lichuan Minority Secondary Vocational School, Laifeng Secondary Vocational Technical School, and Jianshi Secondary Vocational Technical School and obtained the consent of the relevant person in charge of the school. The questionnaires cover secondary vocational schools, high schools, technical colleges, and universities, and the types of schools are relatively comprehensive. The study identified the following criteria for sample selection: (1) The respondent is over 15 years old and under 25 years old; (2) Respondents under the age of 18 need to obtain the consent of the class teacher or their parents before filling out the questionnaire. From May to September 2022, we compiled an electronic questionnaire on the Wenjuanxing platform and distributed the electronic questionnaire to respondents who met the above conditions through WeChat, email, etc. At the same time, we distributed 150 paper questionnaires to respondents at Enshi Vocational and Technical College and Hubei Minzu University. A detailed explanation of the questionnaire’s content and purpose was provided when the questionnaire was distributed and communicated with them through telephone and WeChat.

In this study, we distributed a total of 500 questionnaires and returned 479 questionnaires. As a result of incomplete data filling, 56 invalid questionnaires were eliminated, and 82.8% of them were effective. Based on the data collected, Table 1 indicates the basic personal information and basic family information of the respondents in both schools. Respondents consisted of 58 male students (14.01%) and 356 female students (85.99%). In terms of age structure, there are 60 people aged 15–18, accounting for 14.49%, and 354 people aged 19–25, accounting for 85.51%. From the perspective of education level, most of the respondents have obtained a junior college degree, accounting for 90.34%. In terms of family information, non-single-child families accounted for 85.02%, non-single-parent families accounted for 86.47%, rural families accounted for 66.67%, families with more than 4 people accounted for 82.13%; Most of the family’s annual income was less than 80,000¥, accounting for 83.57%.

**Table 1:** Basic information of the respondents.

Attributes	Items	Frequency	Percent (%)
Gender	Male	58	14.01
	Female	356	85.99
Age	15–18	60	14.49
	19–25	354	85.51
Education	High school (secondary vocational schools)	14	3.38
	Junior college	374	90.34
	College or above	26	6.28
Only child	Yes	62	14.98
	No	352	85.02
One parent family	Yes	56	13.53
	No	358	86.47
Rural or urban residence	Urban	138	33.33
	Rural	276	66.67
Household size	One	4	0.97
	Two	14	3.38
	Three	56	13.53
	Over three	340	82.13
Yearly household income	Below 20,000 ¥	110	26.57
	20,000—40,000¥	90	21.74
	40,001—60,000¥	60	14.49
	60,001—80,000¥	86	20.77
	Over 80,000 ¥	68	16.43

Household size refers to the number of family members living in a household, one refers to one person, two refers to two people, three refers to three people, Over three refers to more than three people.

### 3.2. Measurement

The scale translation method we adopted in this study is based on the suggestion of Brislin (1980). This study used translation and reverse translation methods to translate the scale from English to Chinese, in order to ensure that the translation of each Chinese item in each scale should match the original version as much as possible in terms of concept and language expression, while also meeting the reading cognition of Chinese. First, team members with overseas study experience will translate each item of each scale from English to Chinese. Second, the domestic professors were requested to correct each item in the above translation, and some sentences were slightly changed according to the feedback from the professors. Finally, all Chinese entries are back-translated into English by team members with overseas study experience. During the questionnaire, participants were informed of their level of agreement with each statement of the scale by selecting a number. From 1 to 5 for the family intimacy scale; 1 to 4 for the self-identity scale; 1 to 7 for the psychological capital scale and 1 to 5 for the peer relationships scale. The Likert scale anchor points used in questionnaires were selected in our study. Scores were correlated with adolescents’ family intimacy, self-identity, psychological capital, and peer relationships.

1. Family Intimacy. Olson et al.’s (2013) Family Intimacy and Adaptability Scale was used in this study to measure how family intimacy affects adolescent peer relationships. The scale consists of 16 items, which are self-assessed by adolescents. A sample item is “At home, we all do things together.” The internal consistency coefficient of the family intimacy scale is 0.952 in this study.
2. Self-Identity. As the measure of self-identity in this study, we adopted Ochse and Plug's (1986) self-identity scale. The scale consists of 19 items, which are self-assessed by adolescents. A sample item is, “I am not sure whether something is morally right.” The internal consistency coefficient of the self-identity scale is 0.979 in this study.
3. Psychological Capital. The psychological capital scale used in this study includes four dimensions of self-efficacy, optimism, resilience, and hope. The scale was developed by Luthans et al. (2007). The scale consists of 16 items, which are self-assessed by adolescents. A sample item is, “I can handle many things in my life at once.” The internal consistency coefficient of the psychological capital scale is 0.991in this study.
4. Adolescent Peer Relationships. According to previous studies, peer relationships can be characterized by friendship quality scale (Mounts, 2004; Doumen et al., 2012). This study uses Parker and Asher developed friendship quality scale. The scale consists of 18 items, which are self-assessed by adolescents. A sample item is, “We often get angry with each other.” The internal consistency coefficient of the adolescent peer relationships scale is 0.965 in this study.
5. Control Variable. Some existing studies have shown that personal characteristics of adolescents, such as gender, age, and educational background, have a significant impact on their peer relationships (Adedeji et al., 2022). Meanwhile, family environmental factors such as only child, single parent family, living in rural or urban areas, family size, and yearly family income have a significant impact on adolescent peer relationships (Boele et al., 2019; Livazović and Ham, 2019; Jambon and Malti, 2022). Therefore, controlled variables in the study include gender, age, educational background, only child, single parent family, urban or rural residence, family size, and yearly family income.

## 4. Data analysis

### 4.1. Common method variance

There may be a method bias in this study because all questionnaires were filled out by adolescents themselves (Podsakoff et al., 2003). Testing for common method bias was conducted using the Harman single-factor monitoring method. The first factor can explain 32.459% of the variation after testing and the critical value is less than 40%. Consequently, this study displays no serious common method biases.

### 4.2. Reliability and validity test

In this study, SPSS26 and AMOS24 were used to calculate the composite reliability value (CR), Cronbach’s value, and average variance extraction (AVE) of the relevant data, which were used to test the credibility of the study. In Table 2, all variables have Cronbach’s coefficients greater than 0.9, and all CR values have greater than 0.7. A high level of reliability can be inferred from the scale. The average variance extraction AVE values of all variables were above 0.5. What’s more, there is a high degree of convergent validity and high convincing within all constructs, as their AVE values are greater than 0.5.

**Table 2:** Reliability results.

Factor	Cronbach α	AVE	CR
Family intimacy	0.952	0.553	0.952
Peer relationships	0.965	0.606	0.965
Psychological capital	0.991	0.876	0.991
Self-identity	0.979	0.710	0.979

According to Scott (1995), the chi-square/degrees of freedom ( $\lambda^{2}$ /df), comparative fit index (CFI), Tucker-Lewis index (TLI), root mean square error of approximation (RMSEA)and incremental fit index (IFI)were used to test the model fit. The ideal values are: the $\;\lambda^{2}$ /df is less than 3, CFI, TLI, and IFI are greater than 0.9, and RMSEA is greater than 0.05 (MacCallum et al., 2006). What’s more, compared to other models, the four-factor model’s monitoring indicators have reached the ideal standard, as shown in Table 3. Therefore, all scales in this study have ideal discriminant validity.

**Table 3:** Confirmatory factor analyses.

Model	Factor	χ^2^/*df*	RMSEA	CFI	SRMR	TLI	IFI
Four- factor model	FI，PC，SI，PR	1.288	0.026	0.979	0.035	0.978	0.979
Three- factor model	FI + PC，SI，PR	5.098	0.100	0.695	0.195	0.686	0.696
Three- factor model	FI + SI，PC，PR	5.393	0.103	0.673	0.227	0.663	0.674
Three- factor model	FI+ PR，PC，SI	2.781	0.066	0.868	0.108	0.863	0.868
Two-factor model	FI+ PR，PC+ SI	6.320	0.114	0.604	0.222	0.592	0.605
Two-factor model	FI + SI，PC+ PR	5.393	0.103	0.673	0.227	0.663	0.674
Two-factor model	FI + PC，SI+ PR	5.098	0.100	0.695	0.195	0.686	0.696
Single-factor model	FI + PC + SI+ PR	8.589	0.136	0.436	0.258	0.418	0.437

FI refers to family intimacy; PC refers to psychological capital; SI refers to self-identity; PR refers to peer relationships.

### 4.3. Descriptive analysis

Based on the results of SPSS26, Table 4 shows the standard deviations and correlation coefficients of family intimacy, psychological capital, self-identity, and peer relationships. From Table 4, family intimacy is significant and peer relationships are positively related (*r* = 0.399, *p* < 0.01), family intimacy is significant and psychological capital is positively related (*r* = 0.423, *p* < 0.01). A positive correlation exists between peer relationships and psychological capital (*r* = 0.330, *p* < 0.01). According to these results, this study has preliminarily supported its main hypothesis.

**Table 4:** Means, standard deviations, and interrelations of variables.

	Mean	Std. Deviation	Family intimacy	Friendship quality	Psychological capital	Self-identity
Family intimacy	3.739	0.773	**(0.744)**			
Peer relationships	3.459	0.881	0.399**	**(0.778)**		
Psychological capital	4.575	1.785	0.423**	0.330**	**(0.936)**	
Self-identity	3.061	0.903	0.115*	0.298**	0.138**	**(0.843)**

*p < 0.05, **p < 0.01. The bold values represent the square root of AVE.

## 5. Hypothesis testing

### 5.1. Mediation analysis

Causal stepwise regression (Baron and Kenny, 1986) and Sobel test (Sobel, 1982) were used to test the mediating effect of this study. Following the suggestion of Baron and Kenny (1986), this study uses SPSS26 to examine the influence of family intimacy on adolescent peer relationships, the impact of family intimacy on psychological capital, and the impact of psychological capital on adolescent peer relationships. Reflected from Table 5, model 1a shows control variables explain 1.9% of adolescent peer relationships. Adding the independent variable to model 2a results in a significant regression coefficient between family intimacy and adolescent peer relationships (*β* = 0.459, *p* < 0.001), and the *R*^2^ explanation increase significantly as well (△*R*^2^ = 15.9%, *p* < 0.001), in conclusion, hypothesis 1 is supported. It can be seen from model 3a that the control variables explain 3.1% of psychology capital. In model 4a, after adding the independent variable, the regression coefficient of family intimacy on psychological capital is positive (*β* = 0.979, *p* < 0.001), there is a significant increase in the *R*^2^ explanation (△*R*^2^ = 17.6%, *p* < 0.001), hypothesis 2 is therefore supported. To examine the effects of family intimacy and psychological capital on adolescent peer relationships, model 6a simultaneously introduces both into the regression. After introducing psychological capital to compare model 2a and model 6a, the relationship between adolescent peer relationships and family intimacy has decreased in both coefficient and significance (*β* = 0.459, *p* < 0.001; *β* = 0.370, *p* < 0.001). Therefore, family intimacy and psychological capital have significant effects on adolescent peer relationships. Meanwhile, the variance explained by family intimacy and psychological capital on adolescent peer relationships in model 6a (△*R*^2^ = 0.186) increased significantly compared to model 2a (△*R*^2^ = 0.159). Additionally, the Sobel test showed that the *Z-*value was 3.399, *p* < 0.001, indicating that a mediating effect existed (Sobel, 1982). In other words, adolescent peer relationships and family intimacy are mediated by psychological capital, which supports hypothesis 4.

**Table 5:** The mediating role of psychological capital.

Variable	Peer relationships		Psychological capital		Peer relationships	
	Model 1a	Model 2a	Model 3a	Model 4a	Model 5a	Model 6a
Constant	3.786***	2.249**	7.892***	4.613***	2.525*	1.918*
Gender	−0.321*	−0.285*	−0.561*	−0.484*	−0.231	−0.240*
Age	0.075	0.063	0.295	0.269	0.028	0.038
Education	0.019	−0.072	−0.414	−0.609*	−0.034	−0.017
Only child	0.182	0.214	0.116	0.185	0.163	0.197
One parent family	−0.012	0.018	−0.199	−0.133	0.019	0.031
Rural or urban residence	−0.025	0.048	−0.336	−0.181	0.029	0.064
Household size	−0.054	−0.076	−0.123	−0.172	−0.034	−0.061
Yearly household income	0.013	0.019	0.053	0.065	0.005	0.013
Family intimacy		0.459***		0.979***		0.370***
Psychological capital					0.160***	0.091***
*R* ^2^	0.019	0.178	0.031	0.207	0.121	0.205
△*R*^2^	0.019	0.159***	0.031	0.176***	0.102***	0.186***
*F*	0.999	9.745***	1.613	11.710***	6.175***	10.418***

*p < 0.05, **p < 0.01, and ***p < 0.001.

### 5.2. Moderation analysis

This study tested moderating effects of self-identity using SPSS26, considering reinforcing the influence of the independent variable on the dependent variable through the moderating variables (Baron and Kenny, 1986). To avoid multicollinearity problems, three variables including family intimacy, psychological capital, and self-identity were centered. Next, this study takes the adolescent peer relationships as the dependent variable, adding control variables, family intimacy, self-identity, and the interaction (family intimacy * self-identity) in turn, to test the moderating effect of self-identity on family intimacy on adolescent peer relationships. Also, this study tested how self-identity modifies the relationship between psychological capital and adolescent peer relationships. As shown in Table 6, in model 3b, the interaction (family intimacy * self-identity) has a significant positive impact on adolescent peer relationships (β = 0.222, *p* < 0.001), indicating that self-identity has a moderating effect on family intimacy and adolescent peer relationships, the hypothesis 5 is therefore supported. Thus, family intimacy will have a greater influence on peer relationships for adolescents with high self-identity. In model 3c, the product of psychological capital and self-identity has a significant positive impact on adolescent peer relationships (β = 0.263, *p* < 0.001), indicating that the influence of psychological capital on adolescent peer relationships is moderated by self-identity.

**Table 6:** The moderating effect of self-identity.

Variable	Peer relationships			Peer relationships		
	Model 1b	Model 2b	Model 3b	Model 1c	Model 2c	Model 3c
Constant	3.965^***^	3.853^***^	3.700^***^	3.264^***^	3.280^***^	3.269^***^
Gender	−0.285^*^	−0.267^*^	−0.196	−0.231	−0.220	−0.107
Age	0.063	0.118	0.086	0.028	0.088	0.054
Education	−0.072	−0.071	−0.044	0.085	0.072	−0.011
Only child	0.214	0.178	0.157	0.163	0.131	0.061
One parent family	0.018	0.029	0.017	0.019	0.029	0.021
Rural or urban residence	0.048	0.057	0.048	0.029	0.038	0.036
Household size	−0.076	−0.077	−0.70	−0.034	−0.039	0.000
Yearly household income	0.019	0.007	0.003	0.005	−0.006	0.024
Family intimacy	0.459^***^	0.426^***^	0.382^***^			
Psychological capital				0.160^***^	0.142^***^	0.137^***^
Self-identity		0.247^***^	0.294^***^		0.251^***^	0.372^***^
Family intimacy*Self-identity			0.222^***^			
Psychological capital*Self-identity						0.263^***^
*R* ^2^	0.178	0.241	0.270	0.121	0.185	0.418
△*R*^2^	0.159^***^	0.062^***^	0.029^***^	0.102^***^	0.064^***^	0.233^***^
*F*	*9.745* ^***^	12.768^***^	13.494^***^	6.175^***^	9.119^***^	26.247^***^

Significance levels: *p* < 0.001 (marked as***), *p* < 0.01 (marked as **), *p* < 0.05 (marked as *).

Furthermore, a moderated mediating effect is tested in this study using the SPSS PROCESS plug-in model 14. The PROCESS operation can obtain the moderated mediation effect index and the mediation effect under different values of the moderator variable (Hayes, 2017). Adolescent peer relationships are significantly affected by family intimacy indirectly through psychological capital. As shown in Table 7, the index of moderated mediating is 0.237, and the CI is [0.168, 0.316], excluding 0, indicating that the mediated role of psychological capital in family intimacy and adolescent peer relationships are moderated by self-identity, thus Hypothesis 7 is supported. When self-identity is at a high level, the effect of family intimacy on adolescent peer relationships through psychological capital is 0.366, the standard error is 0.524, and the CI is [0.269, 0.477], excluding 0, indicating that the moderated mediating effect is verified. The effect of family intimacy on adolescent peer relationships via psychological capital is −0.062, the standard error is 0.035, and the CI is [−0.131, 0.007], including 0 when self-identity is low, indicating that the effects were not significant.

**Table 7:** The moderated mediating effect.

	Effect	SE	Confidence interval 95%	
			LLCI	ULCI
Index of moderated mediating	0.237	0.037	0.168	0.316
High SIS	0.366	0.524	0.269	0.477
Low SIS	−0.062	0.035	−0.131	0.007

**Figure 2:**
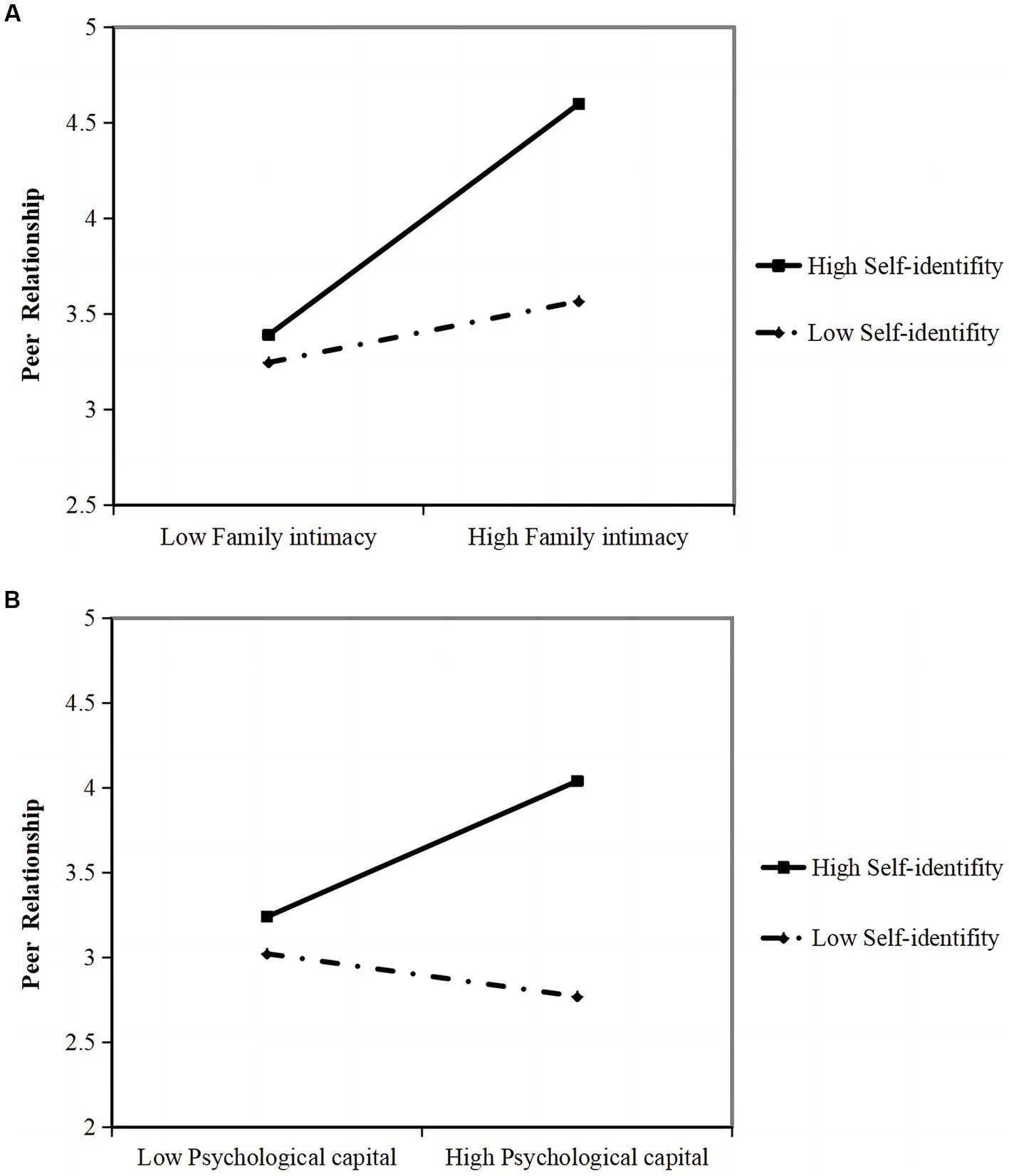
**(A)** Moderating effect of self-identity on relationships between family intimacy and adolescent peer relationships. **(B)** Moderating effect of self-identity on relationships between psychological capital and adolescent peer relationships.

Drawing the moderating effect diagram makes it easy to assess the adjustment effect. From Figure 2A, according to the moderation effect graph, there is a trend to the upper right, which indicates that family intimacy positively influences peer relationships among adolescents. In addition, compared with adolescents with lower self-identity, adolescents with higher self-identity have a higher quality of peer relationships. Figure 2B shows that psychological capital has a greater influence on peer relationships when adolescents have a strong sense of self-identity. Adolescent peer relationships are not significantly influenced by psychological capital in adolescents with low self-identity.

### 5.3. Robustness analysis

In addition, structural equation modeling was used to test the robustness of hypotheses 1, 2, and 3, and the bootstrap method was used to test the robustness of the mediating effect (Hypothesis 4). The result of Amos24 calculation shows, $\lambda^{2}∕df=1.349,$ RMSEA = 0.029, SRMR = 0.032, GFI = 0.865, NFI = 0.933, RFI = 0.930, IFI = 0.982, TLI = 0.981, CFI = 0.982. According to Scott (1995), the structural model has a good fit. The path analysis indicated that family intimacy positively affects adolescents’ peer relationships (*β* = 0.424, *p* < 0.01) and psychological capital (*β* = 1.095, *p* < 0.01), and psychological capital positively affects adolescent peer relationships (*β* = 0.098, *p* < 0.01), which verifies Hypothesis 1, 2, and 3. Following the suggestion of Hayes (2017), bias correction and non-parametric percentile bootstrap method were used to test hypothesis 4. AMOS 24 software was used based on the bootstrap method, a 95% confidence interval was set, and the sampling was repeated 5,000 times. If the confidence interval does not include 0, then the indirect effect is significant. As shown in Table 8, the CI of Bias-Corrected and Percentile of the direct effect and indirect effect does not include 0 in the 95% confidence interval, and *Z* > 1.96 (Shrout and Bolger, 2002), hypothesis 4 has been validated again.

**Table 8:** Robustness analysis.

Effect	Point estimate	Product of coefficients	Confidence interval 95%				
			Bias-Corrected			Percentile	
		SE	*Z*	Lower	Upper	Lower	Upper
Indirect effect	0.107	0.038	2.186	0.039	0.189	0.037	0.188
Direct effect	0.424	0.079	5.367	0.270	0.583	0.265	0.579
Total effect	0.531	0.070	7.586	0.394	0.671	0.394	0.671

## 6. Discussion and conclusion

**Figure 3:**
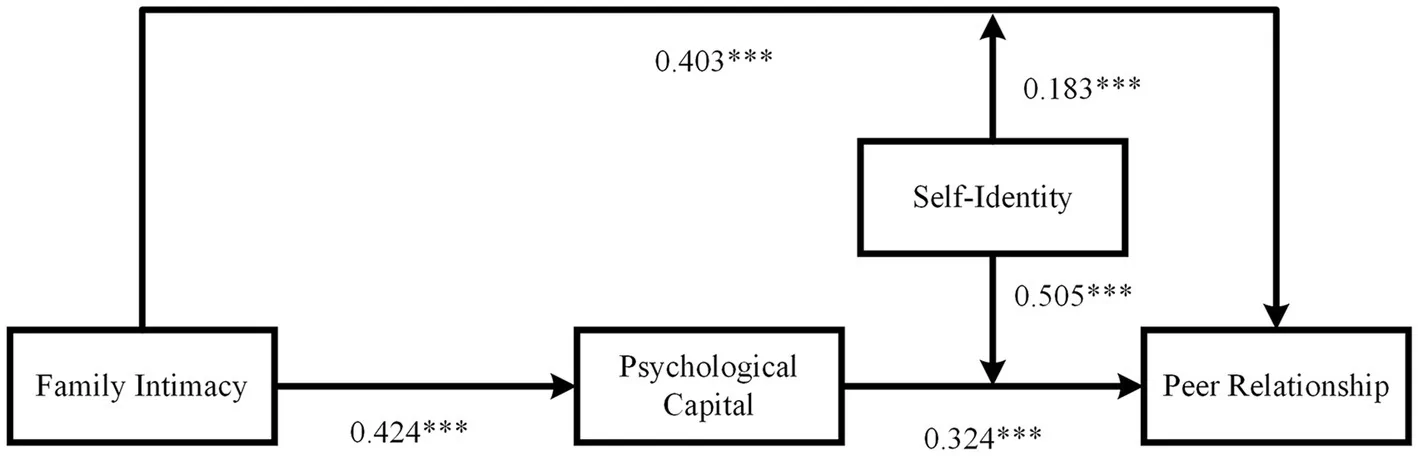
Full model path analysis results.

This research investigates the internal mechanism and boundary conditions of the influence of family intimacy on adolescent peer relationships from the perspective of ecological systems theory. As a result (Figure 3), family intimacy positively affects adolescent peer relationships. Existing research examines the impact on adolescent peer relationships from the perspective of father, mother, parent, or sibling behavior (Boele et al., 2019). Few scholars regard the family as a whole to examine its impact on adolescent peer relationships. Psychological capital partially mediates the relationship between family intimacy and adolescent peer relationships. Similar to previous research conclusions, psychological factors are an important mechanism for the external environment to affect adolescent peer relationships (Sun et al., 2020; Bai et al., 2021; Gao et al., 2022). Family intimacy and adolescent peer relationships were moderated by self-evaluation. Existing research has confirmed that the establishment of peer relationships in specific environments is influenced by individuals’ varying levels of self-identity (Wang et al., 2019; Rizki and Keliat, 2021). It is also confirmed that different degrees of self-identity, as moderator variables, also have differences in the impact of family intimacy on adolescent peer relationships. The direct effect of psychological capital and adolescent peer relationships is positively moderated by adolescent self-identity. In addition, self-identity can positively moderate the effect of family intimacy on adolescent peer relationships through psychological capital. Specifically, when adolescents’ self-identity is stronger, the mediation effect of family intimacy on adolescent peer relationships through psychological capital is stronger. It has not been demonstrated that adolescents with a low sense of self-identity can positively moderate the influence of family intimacy on adolescent peer relationships through psychological capital. The possible reason for this situation is that the research background of this study is based on the Chinese background, and there is a certain degree of cultural background different from the previous research. When self-identity is low, their psychological capital may also be low, so when self-identity is low, the moderated mediation effect does not hold.

### 6.1. Theoretical implications

There are several ways in which our research contributes to the literature. The first finding of this study is that family intimacy impacts adolescent peer relationships and it provided us with a new research direction to deepen our exploration of the mechanisms of family intimacy and adolescent peer relationships. Findings suggest that family intimacy has an impact on adolescent peer relationships. On the one hand, empirical studies have shown that positive, high-quality parent–child communication in the family context is conducive to the establishment and development of parent–child emotional bonding, adolescent social cognition, and interpersonal relationships. On the other hand, studies have indicated that close family intimacy and adaptability play a positive role in adolescents’ emotional and psychological conditions (Waldinger and Schulz, 2016).

The second finding of this study introduces psychological capital as a mediating variable, which enriches the literature research on adolescent peer relationships (Tu, 2020). This research demonstrates that the impact of family intimacy on adolescent peer relationships can be significantly transmitted through psychological capital (Rizki and Keliat, 2021). Psychological capital contains four characteristics. When the four characteristics are combined, individuals will be able to grow and develop more positively as a result (Bea and Chang, 2015). Based on the ecological systems theory, the growth and development of an individual are actively connected with the surrounding environment. Additionally, the study found that close family intimacy can enhance adolescents’ psychological safety and stability, strengthen the connection between adolescents and the surrounding environment, and thus enhance adolescents’ psychological capital. Furthermore, psychological capital plays a partial mediating role in family intimacy and adolescent peer relationships, indicating that there are other mechanisms for the impact of family intimacy on adolescent peer relationships.

The third finding is that the moderating effect of self-identity was also examined in this study. Although family intimacy can enhance adolescents’ levels of adolescent peer relationships by providing a supportive environment, the magnitude of its effect is usually moderated by personal situations, like self-identity. Individuals with different levels of self-identity have different interpretations of family intimacy; Individuals with high self-identity tend to interpret family Intimacy in a positive way compared to individuals with low self-identity (Dugarova et al., 2020). Self-identity is a positive individual difference and it can mitigate the impact of an adverse environment on psychological capital, which is indirectly verified by the research results.

### 6.2. Managerial implications

According to the research results, this study makes some useful suggestions for Chinese, American, and British parents. Suggestions for Chinese parents are as follows: First, parents need to improve parenting styles, create positive emotional relationships, and create a warm family atmosphere. For example, parents should strengthen their correct understanding of family intimacy through schools, communities, and other platforms to strengthen emotional connections between teenagers and family members, so that teenagers can face life with a positive attitude (Shek, 2002). Parents should actively communicate with their children to enhance intimacy among family members. Second, parents should pay attention to caring about their children’s emotional state and guide their children’s cognitive growth. Kerr et al. (2012) indicated that the behavioral control, emotional needs, responsiveness, and social adaptability formed during the growth and development of adolescents are conducive to the smooth establishment of personal peer relationships. Parents should care about their children’s emotional and psychological needs, recognize their children and enhance their positive psychological capital level. Parents should provide their children with active and effective parent–child communication, create a favorable environment suitable for healthy psychological development, and increase their children’s cognitive resources. Finally, parents should create a warm family atmosphere, which is conducive to the establishment of children’s sense of belonging to the family. Parents should strengthen family emotional bonds, provide emotional support for their children, and communicate with them sincerely and equally. It will lead children to have greater extroversion and stability in the face of stress.

Suggestions for British and American parents are as follows: First, during adolescence, children should be encouraged to establish positive peer relationships, which is conducive to the establishment of adolescents’ independence and autonomy, as well as their self-esteem and healthy social emotions (Reed and Trumbo, 2020). Second, parents should support adolescents peer interactions. Peer relationships in adolescence are more prominent. Adolescents’ lack of friends and normal beneficial relationships may lead to increased depression and decreased sense of self-worth (Long et al., 2020). Third, parents should provide their children with love and care, help them establish peer relationships during adolescence, and promote their integration into peer groups, which will help children avoid being troubled by school bullying and isolation. Fourth, parents should value their children and become a person that young people can trust and rely on. On the one hand, this can give adolescents the confidence to talk about their stress and troubles and enable young people to be able to regulate their own stress and negative emotions. On the other hand, it can promote the establishment of adolescents’ positive cognition (Noret et al., 2020).

Due to cultural differences in different countries, family intimacy, and adolescent peer relationships will be different. It is meaningful and necessary to explore more in-depth in combination with cultural backgrounds.

### 6.3. Limitations and future research

Further studies need to be conducted on some limiting factors in this study. First, the measurement of family intimacy in this study only considered the perception of family intimacy by adolescents themselves and did not measure the perception of family intimacy from the perspective of parents. A multilayer model will be constructed for further discussion in future research. Second, this study only considered cross-sectional data and did not consider that adolescents’ cognition will change with age, and continuous tracking can be done in future research. Furthermore, this study did not involve control variables such as different management models of schools, or management methods of head teachers (counselors). In future research, the effects of these control variables should be further investigated by conducting comparative studies between different groups. This study mainly explores the linear relationship between variables, the non-linear relationship between variables is an important direction of future research.

## Data availability statement

The original contributions presented in the study are included in the article/Supplementary material, further inquiries can be directed to the corresponding author.

## Ethics statement

Ethical review and approval was not required for the study of human participants in accordance with the local legislation and institutional requirements. Written informed consent from the participants legal guardian/next of kin was not required to participate in this study in accordance with the national legislation and the institutional requirements.

## Author contributions

XZ and KT developed the conceptual framework, did the data analysis, and wrote the manuscript of the paper. XZ, SQ, and JH collected the data. KT analyzed the data. KT, XZ, SQ, JH, and YN revised the manuscript. All authors contributed to the article and agreed on the final version.

## Conflict of interest

The authors declare that the research was conducted in the absence of any commercial or financial relationships that could be construed as a potential conflict of interest.

## Correction note

A correction has been made to this article. Details can be found at: 10.3389/fpsyg.2026.1955417.

## Publisher’s note

All claims expressed in this article are solely those of the authors and do not necessarily represent those of their affiliated organizations, or those of the publisher, the editors and the reviewers. Any product that may be evaluated in this article, or claim that may be made by its manufacturer, is not guaranteed or endorsed by the publisher.
