# Correction: Family intimacy and adolescent peer relationships: investigating the mediating role of psychological capital and the moderating role of self-identity

**DOI:** 10.3389/fpsyg.2026.1955417

**Published:** 2026-09-04

**Authors:** Xin Zhou, Jin Huang, Sushu Qin, Kangsheng Tao, Yumei Ning

**Affiliations:** 1 School of Humanities and Education, Enshi Vocational and Technical College, Enshi, China; 2 School of Economics and Management, Enshi Vocational and Technical College, Enshi, China; 3 School of Economics and Management, Hubei Minzu University, Enshi, China; 4 School of Business Administration, Zhongnan University of Economics and Law, Wuhan, China; 5 Business School, Yulin Normal University, Yulin, China

**Keywords:** adolescent peer relationships, family intimacy, psychological capital, self-identity, ecological systems theory

In the original manuscript, in section 3.2 (*Measurement*) of section 3 (**Methods and procedures**), the number of items for the Self-Identity scale was incorrectly stated as 4 (it should be 19); the number of items for the Psychological Capital scale was incorrectly stated as 7 (it should be 16); and the number of items for the Adolescent Peer Relationships scale was incorrectly stated as 7 (it should be 18).

A correction has been made to the section [Section 3. Methods and procedures, subsection 3.2. Measurement, paragraphs describing the four scales]:

“2. Self-identity. ...The scale consists of **19** items, which are self-assessed by adolescents...

3. Psychological capital. ...The scale consists of **16** items, which are self-assessed by adolescents...

4. Adolescent peer relationships. ...The scale consists of **18** items, which are self-assessed by adolescents...”

The original version of this article has been updated.

