# Supplementary material for: Family intimacy and adolescent peer relationships: investigating the mediating role of psychological capital and the moderating role of self-identity
Source: Front Psychol. 2023 Jun 29;14:1165830. doi: 10.3389/fpsyg.2023.1165830 (PMC10344464; doi:10.3389/fpsyg.2023.1165830)
Supplement: Supplementary file 2 [file Table_2.DOCX]

Our team is currently researching the impact of family intimacy on student development. Your opinions and suggestions will provide important help to the writing of the thesis. Please express your feelings and experiences as truthfully as possible. Sincerely thank you for your support and cooperation. We will keep your information strictly confidential, and this data will only be used for academic research.

**Part I: Basic personal and family information**

1. Gender

Male

Female

1. Age

15-18

19-25

1. Only child

Yes

No

1. One-parent family

Yes

No

1. Rural or urban residence

Urban

Rural

1. Education

High School（secondary vocational schools）

Junior College

College or above

1. Household size

one

two

three

Over three

1. Yearly household income

20,000 yuan and below

20,000-30,000

40,000-50,000

60,000-70,000

80,000 yuan and above

**Part II: Comprehensive Questionnaire**

1. Family Intimacy (Please choose the one that best suits you from 1, 2, 3, 4, and 5 according to your actual situation. 1-not, 2-occasionally, 3-sometimes, 4-often, 5-always.)

(1) Family members will do their best to support each other in times of difficulty.

1 2 3 4 5

1. Every member of our family is free to express their opinions.

1 2 3 4 5

1. Members of our family are more willing to discuss personal issues with friends than with family members.

1 2 3 4 5

1. Every family member is involved in making major family decisions.

1 2 3 4 5

1. All family members gather for activities

1 2 3 4 5

1. Juniors can express their own opinions on the teachings of elders.

1 2 3 4 5

1. At home, we all do things together.

1 2 3 4 5

1. Family members discuss the problem together and feel satisfied with the solution.

1 2 3 4 5

1. Family members have closer relationships with friends than between family members.

1 2 3 4 5

1. In the family, we take turns sharing different chores.

1 2 3 4 5

1. Family members are familiar with each other's close friends.

1 2 3 4 5

1. When the family situation changes, it is easy to change the family's normal life and family rules accordingly.

1 2 3 4 5

1. When family members make decisions on their own, they like to discuss with family members.

1 2 3 4 5

1. When there are conflicts in the family, members make mutual humility and compromise.

1 2 3 4 5

1. In our house, entertainment is done by the whole family.

1 2 3 4 5

1. Children's suggestions are accepted when solving problems.

1 2 3 4 5

2. Self-identity (Please choose the one that best suits you from 1, 2, 3, and 4 according to your actual situation. 1-not at all, 2-somewhat, 3-mostly, 4-completely.)

(1) I don't know what kind of person I am.

1 2 3 4

1. Others are always changing their opinion of me.

1 2 3 4

(3)I know how I should live.

1 2 3 4

(4)I am not sure whether something is morally right.

1 2 3 4

(5) Most people agree on what kind of person I am.
1 2 3 4

(6)I feel that my lifestyle suits me well.

1 2 3 4

1. My worth is recognized by others.

1 2 3 4

1. I feel freer to be who I am when no acquaintances are around.

1 2 3 4

1. I don't feel like what I'm doing in life is really worth it.

1 2 3 4

1. I think I can adapt to the collective life very well.

1 2 3 4

1. I am proud of who I am.

1 2 3 4

1. People think of me very differently than I think of myself.

1 2 3 4

(13) I feel ignored.

1 2 3 4

(14) People don't seem to accept me.

1 2 3 4

1. I changed my mind about what I wanted from life.

1 2 3 4

1. I don't know what others think of me.

1 2 3 4

1. My feelings about myself have changed.

1 2 3 4

1. I feel that I am acting or doing things for utilitarian considerations.

1 2 3 4

1. I am proud to be part of the society in which I live.

1 2 3 4

1. Psychological capital (Please choose the one that best suits you from 1, 2, 3, 4, and 5 according to your actual situation. 1-not, 2-occasionally, 3-sometimes, 4-often, 5-always.)
2. I believe I can analyze the problem from a long-term perspective and find solutions.

1 2 3 4 5

1. I feel confident about stating things within the scope of study and life.

1 2 3 4 5

1. I can help myself set goals within my learning.

1 2 3 4 5

1. I can communicate and discuss issues with people outside my studies.

1 2 3 4 5

1. I can think of many ways to get out of learning difficulties.

1 2 3 4 5

1. Recently, I feel energetic in achieving the set learning goals.

1 2 3 4 5

1. I strongly believe that there are multiple solutions to every problem.

1 2 3 4 5

1. At the moment, I can be sure of my academic success.

1 2 3 4 5

1. I have difficulty recovering from life setbacks and moving on.

1 2 3 4 5

1. I will try my best to deal with the difficulties encountered in my study.

1 2 3 4 5

1. I can handle academic pressure with equanimity.

1 2 3 4 5

1. I can handle many things in my life at once.

1 2 3 4 5

1. I have positive expectations for uncertain things in life.

1 2 3 4 5

1. I am optimistic about my future life and work.

1 2 3 4 5

1. Things in my current life are not in line with my expected development trend.

1 2 3 4 5

1. I always believe that “after the night comes the light”.

1 2 3 4 5

1. Adolescent peer relationships (Please choose the one that best suits you from 1, 2, 3, 4, and 5 when you're with your companions. 1-not, 2-occasionally, 3-sometimes, 4-often, 5-always.)
2. We always sit together whenever we can.

1 2 3 4 5

1. We often get angry with each other.

1 2 3 4 5

1. He/she told me I was very capable.

1 2 3 4 5

1. My friends and I feel that each other is very important.

1 2 3 4 5

1. When we do things, we always regard each other as companions.

1 2 3 4 5

1. If we are angry with each other, will we discuss together how to calm the anger.

1 2 3 4 5

1. We always discuss our problems together.

1 2 3 4 5

1. My friends make me feel good about some of my ideas.

1 2 3 4 5

1. When I get angry about something, I will tell him/her.

1 2 3 4 5

1. We often argue.

1 2 3 4 5

1. I always play with my friends during recess.

1 2 3 4 5

1. My friends often give me some advice on how to solve problems.

1 2 3 4 5

1. We talked together about things that made us sad.

1 2 3 4 5

1. When we have an argument, it's easy to reconcile.

1 2 3 4 5

1. We often fight.

1 2 3 4 5

1. He/she often helps me to finish the task faster.

1 2 3 4 5

1. We were able to stop arguing quickly.

1 2 3 4 5

1. We often help each other when we do our school assignment.

1 2 3 4 5
